# Research Integrity Among PhD Students at the Faculty of Medicine: A Comparison of Three Scandinavian Universities

**DOI:** 10.1177/1556264620929230

**Published:** 2020-06-12

**Authors:** Bjørn Hofmann, Lone Bredahl Jensen, Mette Brandt Eriksen, Gert Helgesson, Niklas Juth, Søren Holm

**Affiliations:** 1 Norwegian University of Science and Technology, Gjøvik, Norway; 2 University of Oslo, Norway; 3 University Library of Southern Denmark, Odense, Denmark; 4 Karolinska Institutet, Stockholm, Sweden; 5 University of Manchester, UK

**Keywords:** doctoral students, misconduct, integrity, attitudes, knowledge, practice, science ethics

## Abstract

This study investigates research integrity among PhD students in health sciences at three universities in Scandinavia (Stockholm, Oslo, Odense). A questionnaire with questions on knowledge, attitudes, experiences, and behavior was distributed to PhD students and obtained a response rate of 77.7%. About 10% of the respondents agreed that research misconduct strictly defined (such as fabrication, falsification, and plagiarism, FFP) is common in their area of research, while slightly more agreed that other forms of misconduct is common. A nonnegligible segment of the respondents was willing to fabricate, falsify, or omit contradicting data if they believe that they are right in their overall conclusions. Up to one third reported to have added one or more authors unmerited. Results showed a negative correlation between “good attitudes” and self-reported misconduct and a positive correlation between how frequent respondents thought that misconduct occurs and whether they reported misconduct themselves. This reveals that existing educational and research systems partly fail to foster research integrity.

## Introduction

Research misconduct is devastating to research. Research misconduct not only damages the reputation of research and wastes time and money (Martinson et al., 2005) within health sciences, it may also harm patients (Antonelli & Sandroni, 2013; National Health Service [NHS], 2013). The prevalence of research misconduct has been estimated to 2% (fabrication and falsification) and 1.7% (plagiarism) in meta-analyses of several surveys (Fanelli, 2009; Pupovac & Fanelli, 2015). Questionable research practices (QRPs), falling outside of fabrication, falsification, and plagiarism (FFP), are estimated to be even more prevalent; up to 34% in a meta-analysis of surveys (Fanelli, 2009).

Severe research misconduct (such as FFP) and QRP are fundamental challenges, thus, there is broad agreement that research integrity is crucial for the quality of and trust in research (Bosch et al., 2012; Forsman, 1999; Neill, 2006). The European Code of Conduct for Research Integrity defines the fundamental principles of research integrity as reliability, honesty, respect, and accountability (The European Code of Conduct for Research Integrity [ALLEA], 2017). Early career researchers play a crucial role in forming future knowledge production, research environments, and in cultivating the credibility of science. Training and mentoring of researchers from the very start of their career are imperative means to foster and protect research integrity (Fanelli et al., 2015; Krstić, 2015; McNiff, 2006). To support the production of trustworthy research and to nurture research environments of high integrity, training in responsible conduct of research is essential (Kretser et al., 2019). For the purpose of tailoring courses in responsible conduct of research, it is essential to have knowledge about the experiences and attitudes of early career researchers in relation to good and bad research practices. Several survey studies of research integrity among medical PhD students have been carried out in recent years in Scandinavia, providing us with relevant information about knowledge and actions that relate to research integrity (Hofmann et al., 2013, 2015; Holm & Hofmann, 2018; Jensen et al., 2018).

More knowledge is, however, needed, to guide effective actions and to foster research integrity. We particularly need to know whether increased attention and efforts to improve research integrity is effective in cultivating integrity among early career researchers, increasing their knowledge of responsible conduct of research, and of how to act according to the rules.

Accordingly, this article sets out to address four specific questions about research integrity among PhD students at three major medical faculties in the Scandinavian countries:

**Question 1: Knowledge and perception**: What do PhD students know about research misconduct in their area, and how do they perceive the prevalence of misconduct?
**Question 2: Experiences:** Do they experience pressure toward research misconduct, and if yes, what kind of pressure do they experience?
**Question 3: Attitudes:** What are the attitudes of PhD students with respect to various kinds of behavior, considered in the literature to be incompatible with research integrity?
**Question 4: Behaviors**: What kinds of research misconduct do PhD students report to have engaged in themselves?

We also, where possible, investigate the relationships between these four aspects. Misconduct was defined as “behavior by a researcher, intentional or not, that falls short of good ethical and scientific standards” (Kakuk, 2009), and the various types of misconduct were specified in the questionnaire. This definition encompasses both severe research misconduct such as FFP and QRPs.

## Methods

A three-page questionnaire with questions on knowledge about, experiences of, attitudes toward, and behavior with respect to various forms of scientific misconduct was applied. The questions on knowledge, experiences, and actions stem from a questionnaire developed at the Department of Medical Ethics in Lund, Sweden (Nilstun et al., 2010), while the questions on attitudes stem from a recently validated questionnaire originally developed by Kalichman (Holm & Hofmann, 2017; Kalichman & Friedman, 1992). A questionnaire with these elements has been used previously in studies in Norway, Sweden, and Denmark (Hofmann et al., 2013, 2015; Hofmann & Holm, 2016; Jensen et al., 2018). In addition, an instrument relating to behavior were adapted from a Dutch study (Bouter et al., 2016; Tijdink et al., 2016) in order to broaden the range of behaviors investigated beyond FFP. The questions on perception of the frequency of misconduct, the chance of being discovered, and the consequences of being discovered are new.

The invited participants were post-graduate students enrolled in the PhD program at the Faculty of Medicine, Karolinska Institutet (KI, Sweden), at the Faculty of Health Sciences (Department of Clinical Research and Department of Regional Health Research), University of Southern Denmark (SDU, Denmark), and at the Faculty of Medicine, University of Oslo (UiO, Norway). In Norway and Sweden, the questionnaire was distributed, printed on paper to PhD students attending basic, compulsory courses in research methodology, philosophy of science, and research ethics in the academic year 2018/2019. In Denmark, the questionnaire was distributed online to all PhD students enrolled between June 1, 2017 and November 30, 2018.

Participation was voluntary and data were collected and analyzed completely anonymously, which was emphasized both in the introduction letter and when distributing the questionnaires. The questionnaire did not contain any questions about name, age, residential address, or other information that could potentially identify individuals. Furthermore, printed questionnaires were collected in such a way that no researcher could tie specific respondents to filled-in questionnaires (Norway and Sweden) ensuring complete anonymity. In Denmark, a link to the electronic questionnaire (SurveyXact) was distributed by mail with an invitation to participate in the survey, on December 7, 2018, with 2 weeks for completion. Two follow-up emails were sent out to nonrespondents, and an encouragement to participate was included in staff newsletters. Data collection was closed on January 7, 2019.

The data were analyzed using standard statistics functions in SPSS 24. Nominal data were analyzed with χ^2^-test and Fisher’s Exact test, while ordinal data were analyzed with Kruskall-Wallis test. Correlation between ordinal data was tested with Spearman-rho test.

No personal data traceable to individual participants were registered, and therefore the study was not subject to REC/IRB approval, in accordance with the laws in Scandinavia. The participants consented to participating in the study by completing the questionnaire. The questions of the survey are reported to the Norwegian Center for Research Data (Project number 55147).

## Results

The questionnaire was distributed to a total of 367 PhD students and 285 responded. This gives an overall response rate of 77.7%. Table 1 shows the details of distribution and responses as well as demographical data for the respondents of the survey. The gender balance is somewhat different at the three universities but reflects the proportion of the enrolled PhD students. More PhD students at KI (Sweden) do basic research than at the involved departments at SDU (Denmark) and at UiO (Norway). Also, the respondents at KI (Sweden) have more research experience than those at the other two universities. In Denmark and Norway, most of the students had their undergraduate studies in these countries while more students in Sweden had studied elsewhere.

**Table 1.** Response Characteristics and Demographical Data.

Item	Category	KI Sweden	SDU Denmark	UiO Norway
Distributed questionnaires		122	104	141
Returned questionnaires		104	64	81
Response rate (%)		85.2	61.5	57.4
Gender	Female (%)	51.0	69.4	60.5
	Male (%)	49.0	30.6	39.5
Kind of research	Clinical Research (%)	30.1	76.6	67.9
	Basic Research (%)	57.3	9.4	19.8
	Other Research (%)	12.6	14.1	12.3
Time since beginning the PhD	Less than or equal to 1 year (%)	35.6	67.2	76.5
	1–2 Years (%)	51.9	28.1	16.0
	More than 2 years (%)	12.5	4.7	7.4
Lectures or courses in science ethics as an undergraduate	Yes (%)	58.8	71.9	70.0
	No (%)	32.4	12.5	20.0
	Can’t remember (%)	8.8	15.6	10.0
Location of undergraduate studies	Norway (%)	0	0	67.9
	Sweden (%)	45.1	0	4.9
	Denmark (%)	2.0	87.5	1.2
	Elsewhere (%)	52.9	12.5	25.9

*Note.* KI = Karolinska Institute; SDU = University of Southern Denmark; UiO = University of Oslo.

### Knowledge

The PhD students’ knowledge about any occurrence of misconduct at their own department is shown in Table 2. The results show that more PhD students in Sweden know of research misconduct than in Denmark and Norway, but the difference is statistically significant only for the last question (whether they know about anyone at their department that had presented results in any other misleading way than FFP). For this question, the Swedish responses differ significantly from both the Danish and the Norwegian responses.

**Table 2.** Knowledge About Misconduct at Own Department During the Last 12 Months.

Do you know about anyone at your department who during the last 12 months has^a^	KI Sweden (%)	SDU Denmark (%)	UiO Norway (%)
Fabricated data?	4.8	1.6	1.3
Falsified data?	2.9	1.6	1.3
Plagiarized (in any way)?	1.9	0	1.4
Presented results in some other misleading way?^*^	9.6	3.2	5.5
*N*	104	63	75

*Note.* KI = Karolinska Institute; SDU = University of Southern Denmark; UiO = University of Oslo.

a The number given refer to the percentage (%) of respondents answering “yes” to the questions.

* *p* < .05.

### Experiences

When asked whether they themselves during the last 12 months had been object of pressure to fabricate data, 1.9% of the respondents in Sweden answered yes, but 0% in Denmark and Norway. None of the respondents in either country reported to have experienced pressure to falsify data, plagiarize data, or plagiarize publications at any of the universities. However, 2.9%, 1.6%, and 1.3% reported having been the object of pressure to present results in some other misleading way during the last 12 months in Sweden, Denmark, Norway, respectively. Table 3 shows responses to whether the PhD students had experienced pressure to commit other kinds of misconduct during the last year. Although there are differences between the three universities, none of these are statistically significant.

**Table 3.** Experienced Pressure to Commit Misconduct.

Have you during the last 12 months been exposed to unethical pressure concerning	KI Sweden (%)	SDU Denmark (%)	UiO Norway (%)
Inclusion or order of authors?	20.2	14.3	18.2
Design/method?	1.9	3.2	1.3
Analysis?	1	3.2	5.1
Results?	2.9	0	1.3
*N*	103	63	75

*Note.* KI = Karolinska Institute; SDU = University of Southern Denmark; UiO = University of Oslo.

When asked whether they had experienced any consequences of scientific misconduct, about 10% had experienced this. When asked about what kind of consequences, about 3% to 5% reported to have experienced ethical consequences, 0% to 3% had experienced legal consequences, 1% to 2% had experienced methodological consequences, and 2% to 8% had experienced other consequences. There were no statistically significant differences among the universities.

### Attitudes

Table 4 shows the attitudes of the PhD students toward behavior and responsibilities that in the literature are described as undermining research integrity. A total of 65% to 80% of the respondents agree that it is unacceptable to try a variety of different methods of analysis until one is found that yields statistically significant results, while 8% to 13% agree that it is acceptable to omit results to expedite publication, or to falsify or fabricate data to expedite publication (7%–11%).

**Table 4.** Attitudes to Research Misconduct and Responsibilities.^a^

Statement	KI Sweden (%)	SDU Denmark (%)	UiO Norway (%)
It is never appropriate to report experimental data that have been created without actually having conducted the experiment.	92	88.3	88.5
It is never appropriate to alter experimental data to make an experiment look better than it actually was.	98.1	91.8	97.5
It is never appropriate to try a variety of different methods of analysis until one is found that yields a result that is statistically significant.^b^**	64.7	80.3	71.8
It is never appropriate to take credit for the words or writing of someone else.	95.1	95.1	97.5
It is never appropriate to take credit for the data generated by someone else.	94.1	93.4	93.6
It is never appropriate to take credit for the ideas generated by someone else.	94.1	95.1	97.5
If you were confident of your findings, it is acceptable to selectively omit contradictory results to expedite publication.^b^*	10.1	8.2	13
If you were confident of your findings, it is acceptable to falsify or fabricate data to expedite publication.	6.9	10	11.4
It is more important that data reporting be completely truthful in a publication than in a grant application.	36.6	29.5	31.6
If you witness someone committing research misconduct, you have an ethical obligation to act.	88.2	83.3	89.9
If you had witnessed a co-worker or peer committing research misconduct, you would be willing to report that misconduct to a responsible official.^c^*	74.3	60.7	82.3
If you had witnessed a supervisor or principal investigator committing research misconduct, you would be willing to report that misconduct to a responsible official.	70.6	59	75.9
If fabricated data are discovered in a published paper, all co-authors must equally share in the blame.	53.9	42.6	55.7
If fabricated data are discovered in a published paper, all co-authors must receive the same punishment.	38.2	21.3	28.2
*N*	102	61	79

*Note.* KI = Karolinska Institute; SDU = University of Southern Denmark; UiO = University of Oslo.

a The results presented refer to the respondents answering “Agree” or “Strongly agree.” ^b^Comparing KI and SDU. ^c^Comparing SDU and UiO.

* *p* < .05. **p < .005. (Kruskal-Wallis test)

In the responses, writing grant applications is treated differently compared to publications. About one third regard it as more important that data reporting be completely truthful in a publication than in a grant application. Most agree that they have an obligation to act if they discover that someone is committing research misconduct (83%–90%). Slightly fewer respondents report to be willing to act personally by reporting, for instance, a co-worker, principal investigator (PI), or supervisor (60%–82%). Around half of the respondents (43%–56%) think that co-authors must equally share the blame if fabricated data are discovered in a published paper, but less than half (21%–38%) think that all co-authors should receive the same punishment.

From the responses, we calculated the Kalichman 13-item scale (simple summative scale of Items 1–8 and 10–14, Items 7 and 8 reverse scored, range 13–65), which measures the general attitudes to scientific misconduct (Holm & Hofmann, 2017). There was no statistically significant difference between the three universities (Denmark, Norway, Sweden).

Table 5 shows the respondents’ conception of the prevalence of various types of misconduct in their areas of research and their views of the consequences of such behavior. About 10% agree that FFP is common in their area of research, while slightly more agree that other forms of misconduct are common. About 36% to 46% agree that authorship misconduct is common in their area of research. About one third of the respondents think that the risk of being caught for severe misconduct is high, while only 15% to 21% think that the risk of being caught in authorship misconduct is high. The respondents also think that the consequences of being caught correspond to the severity of the misconduct, and only one quarter think that authorship misconduct will result in severe consequences. At all three universities, the prevalence of authorship misconduct was perceived as much higher than other types of misconduct, the risk of detection of authorship misconduct as lower, and the consequences of being detected in having committed authorship misconduct much less severe.

**Table 5.** Perceived Research Misconduct Practices and Consequences.^a^

Statement	KI Sweden (%)	SDU Denmark (%)	UiO Norway (%)
Scientific misconduct such as fabrication, falsification, or plagiarism is common in my area of research.	7.8	8.2	12.7
Other forms of scientific misconduct than fabrication, falsification, and plagiarism is common in my area of research	7.8	13.1	16.5
Authorship misconduct (inappropriate authorship) is common in my area of research	36.3	44.3	46.2
The risk of being detected if you commit scientific misconduct such as fabrication, falsification, or plagiarism in my area of research is high	42.2	31.1	38.5
The risk of being detected if you commit other types of scientific misconduct than fabrication, falsification, or plagiarism in my area of research is high	36.3	27.9	28.2
The risk of being detected if you commit authorship misconduct in my area of research is high	20.6	14.8	21.1
The consequences of being detected if you commit scientific misconduct such as fabrication, falsification, and plagiarism in my area of research are severe (loss of scientific career, loss of funding, retraction of publications)	59.4	41	62.3
The consequences of being detected if you commit other types of scientific misconduct than fabrication, falsification, or plagiarism in my area of research are severe.	48.5	37.7	53.9
The consequences of being detected if you commit authorship misconduct in my area of research are severe	27.7	21.3	28.6
*N*	102	61	78

*Note.* KI = Karolinska Institute; SDU = University of Southern Denmark; UiO = University of Oslo.

a Percent answering “Agree” or “Strongly agree.”

From the responses, we formed three simple summative scales (the “perception scales”). A perceived frequency of misconduct scale (first three items), a perceived risk of detection scale (three next items), and a perceived consequence of detection scale (three last items). All three perception scales have acceptable Cronbach’s Alpha (scores above 0.8).

### Behaviors

When asked if they in the last 12 months had ever fabricated data, only at KI (Sweden) did one respondent (1%) report to have done so. No one (0%) reported to have falsified data or plagiarized data at any of the universities. Only at UiO (Norway) did one respondent (1.3%) report to have plagiarized publications (in whole or in part), but two respondents (1.9%), one respondent (1.6%), and two respondents (2.5%) were uncertain if they had done so in Sweden, Denmark, and Norway respectively. No one reported to have presented data in (some other) misleading way at any of the universities, but 5.9% and 3.9% of the respondents were uncertain if they had done so in Sweden and Norway, respectively.

Table 6 shows respondents’ responses to engage in a broader spectrum of misconduct and QRP throughout the last 3 years. Up to 10% report to have changed data after performing analysis, and up to 14% report to have deleted data before performing data analysis. Up to 10% report to have used phrases or ideas of others without citation at least once. Up to 15% have turned a blind eye to colleagues’ use of flawed data or questionable interpretation of data at least once in the last 3 years. Up to 21% report to have not published parts of a study and up to 36% have added one or more authors who did not qualify for authorship. Up to 18% have reported unexpected findings as having been hypothesized from the start at least once, and up to 20% reported to have decided to exclude data after looking at the impact of doing so on the results. Moreover, up to 28% had decided to collect more data after seeing that the results were almost statistically significant. As shown in Table 6, there are some differences between the three universities.

**Table 6.** Self-Reported Behaviors Within the Last Three Years.^a^

Question: In your work as a scientist, have you engaged in any of the following behaviors in the last three years? (percent at least once)	KI Sweden (%)	SDU Denmark (%)	UiO Norway (%)
Fabricated data?	1.9	0	0
To confirm a hypothesis, selectively deleted or changed data after performing data analysis?^b^*	9.6	0	3.8
Deleted data before performing data analysis?^b^**	13.6	0	6.6
Concealed results contradicting previous research you have published?	2.9	0	0
Used phrases or ideas of others without their permission?	10.6	1.6	12.8
Used phrases or ideas of others without citation?	6.7	6.6	10.4
Turned a blind eye to colleagues’ use of flawed data or questionable interpretation of data?	14.6	9.8	11.5
Modified the results or conclusions of a study under pressure from an organization that (co-) funded the research?	2.9	0	2.6
Not published (part of) the results of a study?^b^*	20.6	6.6	11.5
Deliberately not mentioned an organization that funded your research in the publication of your study?	1	0	2.6
Added one or more authors to a report who did not qualify for authorship (honorary author)?	28.2	36.1	21.8
Selectively modified data after performing data analysis to confirm a hypothesis?	4.8	0	3.8
Reported/ing a downwardly rounded *p* value (e.g., reporting that a *p* value of .054 is less than .05)?	1	0	1.3
Reported an unexpected finding as having been hypothesized from the start?^b,c^**	17.8	3.3	5.1
Decided whether to exclude data after looking at the impact of doing so on the results?^b,c^**	19.6	4.9	5.1
Decided to collect more data after seeing that the results were almost statistically significant?^b,c^**	28.2	1.6	11.5
Omitted a contributor who deserved authorship from the author’s list?	1	3.3	2.6
Stopped collecting data earlier than planned because the result at hand already reached statistical significance without formal stopping rules?	4.9	0	3.8
Deliberately failed to mention important aspects of the study in the paper?	1	1.6	1.3
Not disclosed a relevant financial or intellectual conflict of interest?	1	0	0
Spread results over more papers than needed to publish more papers (‘salami slicing’)?	7.8	3.3	2.6
Used confidential reviewer information for own research or publications?	2.9	0	0
*N*	102	61	78

*Note.* KI = Karolinska Institute; SDU = University of Southern Denmark; UiO = University of Oslo.

a Percent answering that they have engaged in such behavior at least once. ^b^Comparing KI and SDU. ^c^Comparing KI and UiO.

* *p* < .05. **p < .005. (Kruskal-Wallis test)

From the responses in Table 6, we calculated the Research Misconduct Severity Scale (RMSS) scores according to Tijdink et al. (2016). These have a mean of 4.16, 1.20, and 1.95 for KI, SDU, and UiO, respectively. The differences are statistically significant *p* < .0005 for pairwise analysis between KI and SDU and between KI and UiO.

Since there are differences among PhD students at the three universities in length of experience, with KI students having more experience, this might explain the higher RMSS at KI. We therefore analyzed whether the differences in RMSS score among the three universities can be explained by this difference. However, there were no statistically significant differences according to the length of PhD study.

Another possible explanation of the differences in the RMSS score is a difference in student-mix with a larger proportion of KI students doing basic research and fewer doing clinical research. We therefore analyzed whether there are differences in the RMSS score between students doing different kinds of research. The results show that students doing basic research have significantly higher RMSS scores than students identified as doing clinical or other research.

### Correlations Between Attitudes, Perceptions, and Reported Actions

We calculated the correlations between the Kalichman scale and the three perception scales, and the RMSS to investigate the relation between attitudes and experiences and reported actions. We found two statistically significant correlations. One between the Kalichman scale and the RMSS (*R* = −.383, *p* < .0005) and one between the perceived frequency of misconduct scale and the RMSS (*R*= .326, *p* < .0005). There was thus a negative correlation between “good attitudes” and self-reported misconduct, and there was a positive correlation between a perception that misconduct occurs frequently and self-reported misconduct.

## Discussion

About one tenth of the respondents agree that research misconduct (FFP) is common in their area of research, while slightly more agree that other forms of misconduct is common. This can be seen as alarming results, although it comes as little surprise to those familiar with the literature.

Noteworthy in the present study is the finding that a nonnegligible segment of the respondents is willing to fabricate, falsify, or omit contradicting data if they believe that they are right in their overall conclusions. Examples are acceptance of repetition of analysis to obtain statistically significant results (20%–35%), data omission to expedite publication (8%–13%), and falsification or fabrication of data (7%–11%). However, these results are in line with earlier findings (Geggie, 2001; Hofmann, 2016; Hofmann et al., 2013, 2015; Jensen et al., 2018; Okonta & Rossouw, 2014; Rennie & Crosby, 2001). They suggest that the acceptance of actions considered to be misconduct in the literature, when done to promote publication, is not an exceptional attitude among younger researchers. Of course, it cannot be excluded that this is an attitude learnt from more senior members in the research group. The acceptance of incorrect claims seems even more widespread when it comes to what is said in funding applications. Only about one third finds correctness in applications as important as in publications. The findings of a difference in attitude toward reporting on data between grant applications and publications are in line with previous studies (Hofmann et al., 2013, 2015; Jensen et al., 2018). At the same time, most respondents (in several cases around nine out of ten) state that serious instances of misconduct are unacceptable and state that they have a moral obligation to act if they discover research misconduct (83%–90%), which also is in line with previous studies (Hofmann et al., 2013, 2015; Jensen et al., 2018).

Another interesting theme in our results concerns experiences relating to authorship issues. About 40% of the responding PhD students agree that authorship misconduct is common in their area of research: 22% to 36% claim to have been involved in unjustified inclusion of authors themselves, and around 20% report to have felt pressure with respect to the handling of authorship. Only 15% to 21% think that the risk of being caught in authorship misconduct is high, and the consequences if caught are perceived to be less severe than for other types of misconduct. These findings correspond with other studies (Bouter et al., 2016; Tijdink et al., 2016). In comparison, two studies in Sweden addressing recently finished PhD students at medical faculties, showed even higher proportions of PhD students (around 50%) who report to have had at least one paper submitted as part of their thesis that contained undeserving co-authors (Helgesson et al., 2018; Lövtrup, 2010). Around half of the respondents (43%–56%) think that co-authors must equally share the blame if fabricated data are discovered in a published paper, but less than half (21%–38%) think that all co-authors should receive the same punishment.

Moreover, there are differences between the universities in the reporting of knowledge and experience of misconduct in their group or at their department (in Table 6). Although these differences could result from the PhD students at KI (Sweden) having more research experience than those at SDU (Denmark) and UiO (Norway) and thus having had more time to experience and commit misconduct or QRP, this seems to not be the case.

The fact that a worrying number of respondents express willingness to omit contradicting data and even make up fake results to write the paper they want, instead of reporting the results they got, indicates that the present educational and research systems fail in fostering research integrity. It cannot be excluded that these attitudes, in part, are a product of a highly competitive environment where shortcuts appear necessary to survive in the system and to give the impression of progress and success. Hence, it becomes important to influence the general research culture and local research environment in such a way that the honest and constructive attitudes of the majority influence as many colleagues as possible.

### Strengths and Limitations

The main strength of this study is that it brings out experiences and attitudes on issues relating to research integrity among PhD students at medical faculties in Denmark, Norway, and Sweden, some of which are quite striking. Another strength is the comparatively high response rate: The overall response rate of 77.7% is high compared to other studies in this area (Elgesem et al., 1997; Hjellbrekke et al., 2018).

The main limitations relate to the form of inquiry, a questionnaire survey, and perhaps also to how some of our questions are phrased. Occasionally, the reader would probably have benefited from a closer explanation of what certain responses mean to have a better chance of evaluating them. For instance, it may be hard to tell from the bare wording of the question whether not reporting all results is an instance of deceptive behavior, or simply a consequence of journals’ limitations of space, or an active choice of leaving out some less interesting results without being misleading. Moreover, the phrasing whether the respondent had “[u]sed phrases or ideas of others without their permission” may be ambiguous, since researchers may use phrases of others without their permission as long as they cite them properly.

For comparisons between the universities, it would have been preferable to have respondents with equal time as PhD students, even though we have tested for this. The surveys in Norway and Sweden were also distributed on paper, while the survey in Denmark was performed electronically. However, the results do not indicate a format bias.

The difference in the number of responses to the various parts of the study is because not all respondents answered all questions.

The survey was only performed at one faculty at three universities and the results are of course not generalizable. However, they provide important knowledge for comparison and improvement. The survey is requested by other countries and will be applied in several countries in the near future.

## Conclusion

The results of this study show that one tenth of Scandinavian PhD students know about serious breaches to research integrity in their local research environment within the last year. About 1% of the respondents reported to have conducted severe misconduct (FFP) themselves the last year. However, about 10% reported to have plagiarized and about 10% to 20% reported to have altered the data in some questionable way over the last 3 years. Moreover, almost 30% had decided to collect more data to get a statistically significant result for the last 3 years. Also, about one fifth experienced pressure with regard to the inclusion or order of authors and more than one third had themselves added one or more authors who did not qualify for authorship. Learning by being pressured can make you enforce the pressure on the next generation. Hence, there seems to be a self-reinforcing culture with respect to authorship integrity.

In conclusion, Scandinavian PhD students know about breaches with research integrity in their local research environment, and some of them have experienced pressure to commit misconduct. While their reported attitudes are in line with other research findings, they give rise to concern about future research misconduct and urge us to foster good research environments. Moreover, our results imply that existing educational and research systems partly fail to foster research integrity to such an extent that integrity permeates the entire research culture at the studied universities.

## Educational Implications

While necessary, the results indicate that science ethics education and research integrity training are not sufficient for fostering good research integrity. One reason for this can be that strong role models may hamper the effect of good research integrity training programs. Hence, directing educational efforts at supervisors, senior researchers, and scientific role models appear to be an important additional strategy.

## Best Practices

Knowledge about researchers’ attitudes, actions, and knowledge is crucial for targeting improvements. Our study highlighted several areas to improve research integrity, such as authorship. The positive correlation between how frequent respondents thought that misconduct occurs and whether they reported misconduct themselves indicates the importance of fostering good research integrity in research institutions and organizations.

## Research Agenda

The study points at the need for more knowledge about the influence of science ethics education and research integrity training, and the influence of supervisors, senior researchers, and role models as well as the change in knowledge, attitudes, and practices of young researchers by targeting research integrity training of senior researchers.
